# Written products and writing processes in Swedish deaf and hard of hearing children: an explorative study on the impact of linguistic background

**DOI:** 10.3389/fpsyg.2023.1112263

**Published:** 2023-05-09

**Authors:** Moa Gärdenfors, Victoria Johansson

**Affiliations:** ^1^Department of Linguistics, Faculty of Humanities, Stockholm University, Stockholm, Sweden; ^2^Department of Primary Teacher Education, Faculty of Education, Kristianstad University, Kristianstad, Sweden; ^3^Centre for Languages and Literature, Joint Faculties of Humanities and Theology, Lund University, Lund, Sweden

**Keywords:** DHH, CODA, cochlear implant, sign language, keystroke logging, revision, lexical density, writing fluency

## Abstract

The small body of research on writing and writing processes in the group of deaf and hard of hearing (DHH) children has shown that this group struggles more with writing than their hearing peers. This article aims to explore in what ways the DHH group differs from their peers regarding the written product and the writing processes. Participants are all in the age span 10–12 years old and include: (a) 12 DHH children with knowledge of Swedish sign language (Svenskt teckenspråk, STS) as well as spoken Swedish, (b) 10 age-matched hearing children of deaf adults (CODA) who know STS, (c) 14 age-matched hearing peers with no STS knowledge. More specifically we investigate how text length and lexical properties relate to writing processes such as planning (measured through pauses) and revision, and how the background factors of age, gender, hearing and knowledge of STS predict the outcome in product and process. The data consists of picture-elicited narratives collected with keystroke logging. The overall results show that age is a strong predictor for writing fluency, longer texts and more sophisticated lexicon for all the children. This confirms theories on writing development which stress that when children have automatized basic low-level processes such as transcription and spelling, this will free up cognitive space for engaging in high-level processes, such as planning and revision—which in turn will result in more mature texts. What characterizes the DHH group is slower writing fluency, higher lexical density, due to omitted function words, and extensive revisions (both deletions and insertions) on word level and below. One explanation for the last finding is that limitations in the auditory input lead to more uncertainty regarding correct and appropriate lexical choices, as well as spelling. The article contributes with more specific knowledge on what is challenging during writing for DHH children with knowledge of STS and spoken Swedish in middle school, in the developmental stage when basic writing skills are established.

## 1. Introduction

This article explores the written products and writing processes in a group of deaf and hard of hearing (DHH) Swedish children. We particularly want to examine how the children’s linguistic background influence their writing outcome. The group we focus on has proficiency both in spoken Swedish and Swedish sign language (henceforth, STS, Svenskt teckenspråk), and one objective is to identify to what extent the DHH group’s writing performance is on par with peers, and another is to identify areas where the group faces challenges that may be attributed to their linguistic background. We address the impact of the linguistic background through comparisons with hearing peers, with and without sign language knowledge.

We use established methods and measures to describe the linguistic properties of their written texts, and relate them to their writing processes. To our knowledge, no such systematic description previously exists for the DHH group. The article serves the purpose of generally informing of this group’s capacity and challenges in regard to writing development, but also adds to the overall knowledge of writing processes in the developmental span of 10–12 years of age.

Writing is often described as a complex problem-solving activity, and developing writing skills during the school years has been compared to a design process (Maun and Myhill, 2005) where children learn to master the orchestration of several activities in a creative, generative process. How successful the outcome is for an individual child will depend on many things, such as cognitive development, linguistic proficiency, motivational factors, the school system and the specific literacy instruction the child receives (Myhill, 2009). In broad terms, writing development can be studied from two main perspectives: the written product, i.e., the finished written text, and the writing process, i.e., what happened when the text was written, or the study of the unfolding of events and actions that led to the finished text (Berninger et al., 1996).

As Myhill (2009) points out, the linguistic development in general and the writing development in particular is less studied for the age span 11–16 than the years before and after. The reason is probably on the one hand a strong research focus on younger children and their initial experience of learning to write, and on the other hand an interest for older teenagers’ writing ability to meet the demands of higher education or work life when they leave upper secondary school. As a consequence, writing development during the middle years of school deserves more attention, and this article is contributing to filling that gap.

While the writing development of children in general is understudied in this age group it is even more so in regard to DHH children. The DHH group in the present study uses hearing aids (HA) or CI (cochlear implant, that is, an advanced hearing aid), which enable them to hear and develop spoken language. As many DHH children with CI in Sweden, they attend mainstream schools and are taught reading and writing in similar ways as their hearing peers (Socialdepartementet, 2016). However, we also know that as a group, the DHH children, due to their hearing loss, may have received less linguistic input through speech during their childhood, which in comparison to hearing peers may negatively influence their academic achievements later in life (Yoshinaga-Itano, 2003; Arfé, 2015; Mauldin, 2019).

This article aims to address writing from a linguistic point of view, by exploring how the written product is related to writing processes in a group of DHH Swedish children, who are proficient in spoken Swedish and STS.

### 1.1. Theoretical background

In the following background, we outline previous research on linguistic development which is relevant for understanding what characterizes the age span 10–12. We further sketch out the overall writing development in the age span, and expand in particular on what is known of the writing development of DHH children.

Linguistic development during the school age is generally characterized by an increase in text length (see Scott, 1988), vocabulary growth (see McCutchen, 1986; Crossley et al., 2014) and advances of rhetoric aspects, reflected in e.g., deliberate variation of linguistic aspects such as sentence length, or purposeful repetitions of lexical items (see Myhill, 2008). Children are thus expected to develop a set of linguistic skills during the period we are addressing (Truckenmiller et al., 2021). However, cross-linguistic descriptions on the overall linguistic development in the age span 10–12 indicate that the advancement is complex, and does not necessarily demonstrate a linear pattern concerning text length and vocabulary growth (Berman and Verhoeven, 2002).

In the Swedish context, the age span 10–12 years is equivalent to school years 4–6, and the Swedish middle school. At this stage, the children have acquired the basic literacy skills, and are now–according to the school curriculum–expected to use reading and writing as tools for obtaining and develop new knowledge in other subjects. Although increase in text length is repeatedly connected with age and writing development in the literature (see Nippold, 1993), studies report a step-wise, rather than linear pattern (Berman and Verhoeven, 2002). The non-linearity is reflected in a Swedish study comparing 10-year-old children to 13-year-old children (Johansson, 2009) which showed no significant differences in text length in written texts regarding commonly used length measures such as number of words, clauses, or T-units (i.e., a main clause with attached subordinated clauses, Hunt, 1966). This lack of age difference is explained by a substantial variety in text length between participants within each age group. Another study on Swedish children’s text writing compared 11-year-old children (grade 5) to 15-year-old children (grade 9) and reported significant increase in text length (Löhndorf, 2021). This study used texts from the National tests in Sweden, and the topics were thus not comparable between age groups. The age gap between the compared groups was also bigger, which may explain the significant increase in text length. It may be noted that Johansson (2009) also includes comparisons with a group of 17-year-olds (grade 11), and the developmental leap regarding text length was substantial between age 13 and 17.

Two common measures for examining and describing vocabulary growth, or lexical development, are lexical density (i.e., the proportion of content words to the total number of words; see Halliday, 1985) and lexical diversity (i.e., the variation of unique words, as a measure of lexical growth; see Malvern et al., 2004). These measures did not differ between age 10 and 13 in the written texts in the Swedish data described in Johansson (2009), although there were salient differences showing higher lexical density and diversity in equivalent spoken data by the same participants. However, Löhndorf (2021) demonstrates a significant difference between grade 5 and 9 regarding lexical diversity, and a further exploration on the use of adjectives, showed a high adjective density for younger age groups in written texts (grade 3), decreasing in grade 5, and further in grade 9. In addition, an increased lexical sophistication through examining how the types of nominal meaning changes in the age span 9–17 (grade 3, 5, 9, and 11) was reported, as was a complex but consistent expansion of more abstract use of adjectives.

To sum up, previous studies of linguistic development for Swedish children in the age span 10–12, fail to identify consistent developmental trends through quantitative measures targeting text length and vocabulary development. Existing data, however, seems to indicate great individual variation, and it may be that children are practicing and establishing a variety of linguistic skills simultaneously, and that this is one reason why clear developmental trends are difficult to differentiate in this age span. In a prolonged developmental perspective, e.g., between age 9 and 15 (Löhndorf, 2021), or between age 10 and 17 (Johansson, 2009), a significant developmental leap is visible.

#### 1.1.1. Writing development

The general linguistic development outlined above is tightly connected to the writing development, including the evolvement of skills such as planning what content to include in a text, organizing and structuring the content, giving the content a linguistic form—involving making the best lexical choices and structuring phrases, clauses and sentences grammatically—and anticipating and adapting to the needs of a future reader (Kellogg, 2008; Tolchinsky, 2016).

The writing development in the age span 10–12 is characterized by experimenting with punctuation, according to Berman and Verhoeven (2002), who comment that younger children (age 9–10) may omit all punctuation, except perhaps a full stop in the end, while children age 13–14 use a wide range of hyphens, parenthesis, and commas, to comment and expand on content in their written texts. However, consequent use of paragraphs and headers are not yet established. Another observation is that the children demonstrate genre awareness, as shown by 13-year-olds using more genre-typical features (regarding lexicon and syntactic structure) in the expository school genre, and that they make clear distinctions between speaking and writing. Children do not “write as they speak”—illustrated among other things by the lack of discourse markers in writing (while used in abundance in speaking).

Some studies have identified gender as a factor influencing writing outcome, although gender differences are not reported by either Berman and Verhoeven (2002) or Johansson (2009). But other studies have found that girls have more proficient transcription skills (Berninger and Fuller, 1992), and a study of 9-year-old Australian girls report that they generally wrote longer and more complex texts, using a wider range of adjectives and verbs compared to the boys in the same schools Kanaris (1999). Truckenmiller et al. (2021), who investigated the writing of informational texts by 10–15-year-old children in the USA, similarly report that girls consistently outperform boys, regarding both vocabulary and syntax, and suggests that one reason for this is an accumulated effects of skill development for girls. Gender differences are further addressed in a study that examines fluency in writing using keystroke logging (Zhang et al., 2019). Here, children in grade 6–9 in USA wrote argumentative texts, and results demonstrated that girls were more fluent, engaged more in both local and global editing, and paused less during text production. In addition, they scored higher on text quality (see also Al-Saadi, 2020, reporting similar findings from studying writing fluency, but for undergraduate students).

To describe how writing processes unfold with age, we turn to the framework of the seminal cognitive model of writing by Hayes and Flower (1980). They define three main processes: planning, translation/transcription and revision.

Planning has been investigated through the study of pauses: typically their duration and location (Spelman-Miller, 2006). The underlying assumption is that increased cognitive load is mirrored in longer pause durations, and that the location of those will indicate contexts where writers particularly need to reflect. From a developmental perspective this may give insights into what linguistic features (e.g., grammatical structures, or expansions of noun phrases) that a writer is acquiring right now. Pauses are thus seen as indirect evidence of writers’ cognitive activities. Systematic investigation of pauses during writing in young writers have shown that long pauses are common in syntactic boundaries, for instance described by Ailhaud et al. (2016) in a study of narrative and expository texts written by French 9–10-year-olds, 12–13-year-olds, and 14–15-year-olds. A typical finding for all writers is that it is very common to pause between sentences–probably because this is a natural place both to evaluate what has been written and to plan forward (Fayol et al., 2012).

Translating, that is the general ability to put one’s thoughts into words on paper or screen, is a fundamental condition for successful writing development. A prerequisite for writing development to take place would also be the automatization of writing processes, where fluent transcription skills play an important role (Fayol, 1999; Graham and Harris, 2000). Theories about working memory capacity and writing (Just and Carpenter, 1992; McCutchen, 1996) propose that processes compete with each other over the limited cognitive resources, and automatized processes will free up space that can be used for cognitively more demanding tasks. Following this reasoning, inexperienced (often younger) writers who have not yet automatized low-level processes such as transcription and spelling will need to devote more cognitive effort to those, but once they are established, cognitive resources will be freed up, and more attention can be paid to high-level processes. Research suggests that transcription skills are a “bottle-neck” to young writers who has not automatized them (Alves and Limpo, 2015), and also that transcription skills play an essential role for increased spelling accuracy (Limpo et al., 2017).

Finally, the complexity of revision is outlined in Faigley et al. (1981), describing for instance the differences between surface changes and so-called text-based changes. An overview on how revision develops is found in Chanquoy (2001), who summarizes that younger children to a higher degree engage in making changes on linguistic form, on the surface level. However, with age, more attention is given to deep revisions, also described as content revisions, or semantic revisions. A study comparing types of revisions in 10-year-olds and 13-year-olds demonstrated that the percentage revisions concerning spelling remained constant through the age span. However, 10-year-olds engaged more in changes on word level, and 13-year-olds attended to punctuation (Johansson, 2000).

The writing development model by Berninger and Swanson (1994) (see also Berninger et al., 1996) takes the starting point in the writing processes described by Hayes and Flower, and use them in a description of three stages of writing development. The importance of transcription skills is especially emphasized in the model. In the first stage (grade 1–3), low-level processes connected to transcription processes are developed, which includes translation and spelling. Gradually, high-level processes (e.g., planning and revision) evolve, first on a local level, and later (grade 4–6) children will expand their planning and revision to a more global text level. At this age, the transcription skills are also more automatized, which will free up cognitive capacity.

The establishment of this automatization is for instance shown by von Koss Torkildsen et al. (2016) who studied Norwegian 8-year-old children whose narrative text products were compared to their writing processes. Here, children with good reading and spelling abilities revised more, and, in general, revisions occurred locally. This study further included background factors, such as reading and writing skills, and reported that the number of revisions that were made during writing predicted the length of the narratives, and that a higher number of revisions also was associated with better texts. Analyses also revealed that children with good spelling and reading skills made the largest number of online revisions. These findings connect increased revision to more skilled writers.

Descriptions of the general development of writing processes further shows that although Swedish 10-year-olds have typing skills which are less mature than older peers (Wengelin, 2006), they have automatized many of the low-level processes. This includes basic orthographic rules, as well as use of punctuation and grammar (see also Johansson, 2000, 2009).

Younger children’s writing strategies have often been described as linear, where one sentence at a time is added to the text—a writing behavior that in Bereiter and Scardamalia (1987) model of writing development is called knowledge telling. This developmental stage is characterized by local writing strategies where the writer step-by-step adds one piece of information to the previous one in a linear process, often without any re-organization of the ideas once they have been written down. Older and more mature writers would instead be described as knowledge-transformers and adopt more global strategies in their writing, which include planning and revision of the text, often with the purpose to meet the writer’s rhetoric and pragmatic goals. The start of the knowledge-transforming stage is generally associated with the mid-teens.

#### 1.1.2. The DHH group and use of sign language

Today we know that sign language is indispensable for the DHH population, especially when considering that this group, due to their hearing loss, until the breakthrough of advanced hearing technology, to a limited extent has acquired and used spoken language. Also, learning a sign language at school has historically not always been an option for this group. As a result, many DHH children showed severe language delays. Their first contact with a (sign) language was often when they started to attend a boarding school for the deaf and for the first time met and communicated with older deaf classmates (e.g., Svartholm, 1984). One consequence of the delayed start of acquiring language was severe reading and writing delays, which pursued the DHH children across the lifespan and in turn impacted both cognitive development and academical achievements.

In the Swedish case, a collaboration between Swedish researchers and teachers was initiated in the 1980s, and eventually resulted in that sign language courses were provided to younger deaf children and their hearing parents. The outcome was that the signing DHH students’ results soared, and outperformed their peers who learned sign language later in life. The difference was so distinct, that a bilingual curriculum was inserted for all the deaf in Sweden. The understanding of the importance of acquiring sign language early for this group led to that many DHH children in Sweden were offered to learn sign language early in life, along with their parents (Svartholm, 2010).

But when hearing technology, like cochlear implants (CI), had its breakthrough in the beginning of the 2000s, many DHH children and their parents chose to only use spoken language at the expense of sign language. This has resulted in that many DHH children today typically attend schools for hearing, and have hearing friends (Szarkowski, 2018).

Thus, hearing technology, especially cochlear implants, has certainly made a difference regarding literacy skills for many DHH children worldwide, where they often outperform their deaf peers without hearing technology, thanks to that they now can take advantage of sounding strategies in similar ways as their hearing peers. However, they still show delays compared to their hearing peers (e.g., Arfé et al., 2016), and explanations include that there are limitations in how and when the advanced hearing technology can be used. Because of this, the DHH group has restricted access to spoken language compared to their hearing peers. In addition, background noise will impede the DHH group’s ability to listen and comprehend, which is shown in a recent experimental study, that examined listening with different noise conditions in a simulated class-room context. Here, DHH children report perceived listening effort in situations with increased noise (Brännström et al., 2022). The limited auditive access can also cause delays in the use and development of literacy (Yoshinaga-Itano, 2003; Arfé, 2015; Mauldin, 2019). Since there are more DHH children who have none or limited knowledge in a sign language than vice versa today (Socialdepartementet, 2016), it has been argued that children with hearing technology should, in addition to spoken language, also be offered full and early teaching of sign language. The motivation is that since sign language does not rely on hearing ability, it will provide the group with additional linguistic input. This is based on studies that have compared bilingual (signing and speaking) DHH children with monolingual (only speaking) DHH children, and showed, in line with the previous findings of the signing deaf children (without hearing technology), that the signing DHH children with hearing technology also outperformed their monolingual DHH peers in all literacy outcomes, including the speech outcomes (e.g., Hassanzadeh, 2012; Amraei et al., 2017; Goodwin et al., 2017; Caselli et al., 2021; Gärdenfors, 2021; Johnson, 2021).

#### 1.1.3. Writing in children with hearing loss

Previous research of DHH children’s linguistic development has primarily focused on reading and spoken skills, and experts have repeatedly pointed out the need for expanding the research and study writing as well (for reviews, see Williams and Mayer, 2015; Mayer and Trezek, 2018). The limited research on DHH children’s writing proficiency focusses mostly on word level and spelling. A finding related to spelling is that DHH children (regardless of linguistic background) face difficulties with (but do not lack) phonological processing. This has been demonstrated through the examination of more phonologically implausible spelling errors (e.g., words that are not spelled as it sounds). The suggestion is that the DHH group’s insufficient hearing restrain them to use spelling strategies built on visual representations instead (e.g., Bell et al., 2019; Gärdenfors et al., 2019; Daigle et al., 2020). A difference between deaf children without hearing technology and the DHH children who use it, lies in that the latter group can draw on auditory strategies in similar ways as their hearing peers. This includes, for instance, sounding out words in order to write. This approach to spelling can sometimes result in the same kind of sounding-based spelling errors that are recurrent among hearing children (Gärdenfors et al., 2019).

A few earlier studies have focused on examining the writing processes of DHH children and relate them to their writing products (e.g., Asker-Árnason et al., 2010, 2012; Gärdenfors et al., 2019, Gärdenfors, 2021). The overall results show that the DHH group produce shorter texts, demonstrate lower transcription skills (measured as transition times, a measure of how quickly a writer moves from one key to another during writing), and slower writing flow (i.e., the production of fewer words or characters per minute), use more pauses and revise more than their hearing peers. These studies convey that the children in the DHH group who use hearing technology, share some—but not all—characteristics of writing with their hearing and deaf peers (i.e., deaf children without hearing technology). A similarity between the DHH group and their deaf peers is a dependency for visual input to enhance language acquisition, but also a tendency to use fewer grammatical words—something which is manifested through a higher lexical density (Singleton et al., 2004; Asker-Árnason et al., 2012; Gärdenfors, 2021). Additionally, both DHH and deaf children and adults make more morphological errors (e.g., Wengelin, 2002; Bowers et al., 2014; Gärdenfors, 2023).

Studies have further reported that the development of writing may be more challenging for the DHH group than the development of spoken and reading abilities. This assumption is based on that good spoken and reading abilities do not always correlate with the writing outcomes for this group. The explanation for this discrepancy is that the DHH group have more difficulties in transferring morphosyntactic structures from speech into writing (Arfé, 2015; Mayer et al., 2016; Mayer and Trezek, 2018; Breland et al., 2022). Examples of this is reported in Arfé (2015) who compared spoken and written narratives by 42 Italian DHH participants, age 7–15 with 48 age-matched hearing peers. The groups produced equally many words in the spoken condition, but the DHH group had more clauses. In writing, both groups produced just as many clauses, but the DHH group had less words, compared to hearing peers. The author argues that the DHH group has a disadvantage in the writing modality, due to higher cognitive cost of the writing task. Another example of how DHH writers struggle comes from Breland et al. (2022) who examined spoken and written narratives by eight grade students in USA: 51 DHH students and 52 hearing students. Both groups performed similarly on the spoken tasks, but the DHH group was found to have less complex writing than the hearing peers, and also to be less complex in writing than in their own spoken narratives. One conclusion was that the DHH students may have difficulties to meet the writing demands necessary for success in future academic achievements. Other relevant findings from research on writing in the DHH group include Gärdenfors (2023) who explored the writing performance of children with hearing loss in the age span 12–15 in an intervention study aiming to increase narrative skills in writing. The results indicated that the severity of hearing loss did not influence the text quality, and that girls produced written narratives with higher text quality.

#### 1.1.4. The present study

The present study is part of a larger project that addresses writing in the DHH group. In this section we briefly outline the previous findings from the project, and define the scope of this study. The first study examined the spelling of 33 deaf, hard of hearing, CODA (children of deaf adults) and hearing children between the age of 10 and 11 (Gärdenfors et al., 2019), where the most important outcome was that deaf children (i.e., DHH children without hearing technology) used different spelling strategies than the other groups. The study suggested that this was due to that auditory input is totally absent for this group. A final result for all DHH children (i.e., with access to hearing technology and not) is that they engaged more in spelling than their hearing peers. In the second study, Gärdenfors (2021), compared the writing processes and the written products (i.e., finished texts) for 20 bilingual children of STS and spoken Swedish: 10 DHH children and 10 CODA children, age 10–11. Results demonstrated similar outcomes between the groups for the written products, with the exception that the texts by the DHH children had higher lexical density. A conclusion of the study was that the two groups used different writing processes to reach similar final texts. A third study in this project is reported in Gärdenfors (2023), which encompassed 24 DHH writers age 8 to 18. The purpose was to investigate the lexical and syntactic development in DHH children, with different linguistic backgrounds (knowledge of spoken Swedish, knowledge of STS or not). The findings showed that the linguistic development of this group followed a similar developmental trend as their hearing peers, but with a delay of about one year.

The three previous studies have revealed that access to spoken Swedish and knowledge of STS seem to influence both the written products and the writing processes. The present study aims to explore these relationships further, through a more systematic approach to how products and processes are related, were we specifically target revision behavior and pauses as a sign of planning. Particularly, we address how the background factors hearing and knowledge of STS predict writing outcome. To control for this, we compare the DHH group to hearing peers with STS knowledge (the CODA group) and without STS knowledge (the hearing group). We have also aimed at keeping the age span as small as possible.

More specifically, the purpose of the present study is to address writing from a linguistic point of view, by exploring how the written product is related to writing processes in a group of DHH Swedish children in the age span 10–12, who are proficient in spoken Swedish and STS. Following what previous research has identified as typical for this age group, we will investigate how text length and lexical properties relate to writing processes such as planning (measured through pauses) and revision. The DHH participants in our study have knowledge of STS in addition to spoken Swedish, which gives us a unique possibility to explore how proficiency in STS impact their writing. To examine how the DHH group compares to peers, and at the same time explore the impact of hearing as well as STS, we have included age-matched hearing peers in the study: on the one hand a group of hearing peers with no sign language knowledge, and on the other hand a group of CODA children. Since the latter group knows STS, we are provided with a way to compare how linguistic background (in this study: knowledge in STS as well as knowledge in spoken and written Swedish) influence the outcome in writing. Given that previous research has identified age as a strong factor for linguistic development, and that several studies have reported findings that girls tend to write longer and more sophisticated texts, we have also included age and gender in our background analysis, to enhance a better overall understanding for factors influencing the writing development. By exploring the data in this broad way, the expectation is to identify areas that are fruitful to address, for instance with a more hypothesis-driven design, in future studies.

The study is guided by the following research questions:

1. How are written products and writing processes realized and related in narrative writing for a group of 10–12-year-old DHH children, and hearing, age-matched peers with and without STS knowledge?
2. To what extent are the background factors age, gender, hearing, and Swedish sign language (STS) associated with the outcome of the written products for these children?
3. To what extent are the background factors age, gender, hearing, and Swedish sign language (STS) associated with the outcome of the writing processes for these children?

## 2. Materials and methods

### 2.1. Participants and data collection

This study includes 36 students between 10 and 12 years old, collapsed over three groups, depending on their hearing and linguistic background: a group of 12 deaf and hard-of-hearing (DHH) children (which is the focus of this study); a group of 10 children of deaf adults (CODA); and a group of 14 hearing children.

Participants were recruited from all over Sweden through two cochlear implant-clinics who conveyed information about the study to patients and their parents; through the deaf community including several schools for the deaf or schools for hard-of-hearing children; and through mainstreamed schools for hearing children. The main part of the data was collected in the participants’ schools, but, when more convenient, data collection also took place in the cochlear implant clinics, or at the participants’ home. Most data from the CODA group were collected during an annual week-long class offered to CODA children from different schools, where they meet at a school for the deaf, to use and practice STS, and socialize with other same-aged hearing peers who also have deaf parents. Finally, the non-signing hearing children (controls) all come from the same class in a small village in southern Sweden.

The DHH group consists of twelve DHH children between the age of 10 and 12: three boys and nine girls, see Table 1. Seven of the children use hearing aids (HA), and the remaining five children use cochlear implants (CI), i.e., an advanced hearing technology that needs to be operated through the cochlea inside the ear. With help of these hearing technologies, they use and comprehend spoken Swedish. In addition, all but one has fluent signing skills which is demonstrated through their high scores (often close to the maximal score of 4.0) on the sign language test SignRepL2 (see below). The explanation to the overall high-test scores is that ten of the DHH children have fluently signing parents (many of them are deaf). Only two parents did not master, or had very limited knowledge in STS.

**TABLE 1:** Background information for the participants: the group of deaf and hard of hearing children (DHH), the children of deaf adults (CODA) and the hearing children (Hearing).

	Age	Gender	Sign-RepL2-score	Signing parents	Hearing technology	Age at 1st implant	Age at 2nd implant
**DHH**	10.7	Boy	3.88	Yes	HA*		
	10.7	Girl	2.44	No	CI**	2 year	3 year
	11	Girl	3.54	Yes	CI	1 year, 2 month	2 year, 1 month
	11.1	Girl	3.86	Yes	CI	1 year, 6 month	n/a
	11.3	Girl	3.78	Yes	CI	9 month	9 month
	11.4	Girl	3.8	Yes	CI	2 year, 2 month	4 year, 5 month
	11.6	Boy	3.84	Yes	HA		
	12	Girl	3.98	Yes	HA		
	12.7	Boy	3.68	No	HA		
	12.8	Girl	3.98	Yes	HA		
	12.8	Girl	3.96	Yes	HA		
	12.9	Girl	3.92	Yes	HA		
**CODA**	10.9	Girl	3.92	Yes			
	11	Boy	3.7	Yes			
	11	Boy	3.78	Yes			
	11	Boy	3.92	Yes			
	11.2	Girl	3.84	Yes			
	11.3	Girl	3.62	Yes			
	11.4	Girl	3.44	Yes			
	11.6	Girl	3.76	Yes			
	11.7	Girl	3.46	Yes			
	12.5	Girl	3.52	Yes			
**Hearing**	10.3	Girl	1.96	No			
	10.3	Girl	1.76	No			
	10.4	Girl	2.38	No			
	10.5	Girl	2.02	No			
	10.6	Girl	2.06	No			
	10.6	Girl	2.2	No			
	10.7	Boy	1.94	No			
	10.8	Boy	2.06	No			
	10.9	Boy	2.08	No			
	11.1	Girl	2.58	No			
	11.2	Boy	2.2	Yes			
	11.4	Girl	1.98	No			
	11.5	Girl	2.24	No			
	11.6	Boy	2.1	No			

*HA, hearing aids; **CI, cochlear implant.

The CODA group consists of ten children of deaf adults between the age of 10 and 12: three boys and seven girls. They are hearing but have deaf and signing parents, which explains why they master both spoken Swedish and STS fluently. Their fluent signing knowledge is also confirmed through their high scores on SignRepL2 reaching between 3.5–3.9 points.

The hearing group consists of fourteen children between the age 10 and 11: five boys and nine girls. They all have typical hearing but no signing knowledge. The hearing group includes one boy with signing (but hearing) parents, because he has a deaf sibling, but he does not master STS himself.

### 2.2. SignRepL2, Swedish sign language test

This study includes STS knowledge as a predictor, and for this reason the Swedish sign language repetitive test, SignRepL2, was given to all participants. In the test, which was initially developed for L2 learners of STS (Schönström and Holmström, 2017), participants were asked to watch and imitate fifty short STS sentences as exactly as possible. Being able to imitate more advanced features of Swedish sign language distinguishes a skilled signer from a novice and/or a L2-signer. The SignRepL2 test takes 10 min to administrate and consists of a 0–4 points scale. Reaching the four points ceiling represent fluent signing skills. Reaching around two points corresponds often to no, or limited knowledge in STS (Holmström, 2018; Schönström et al., 2021).

### 2.3. Equipment

The written texts were collected by means of keystroke-logging, using ScriptLog (Frid et al., 2014). The program looks like a simple word processor, without spellchecker, and records the keystroke and mouse movements, give them a time stamp, and creates a temporal log of the writing activities. ScriptLog allows for the recording of writing processes in an unintrusive way, while also providing the researcher with the possibility of replaying the writing session, and extracting output for further analyses of such things as overall descriptive statistics on writing time and number of written and removed characters, pause duration and location, and revisions. The output from ScriptLog can further be exported to another keystroke logging program, Inputlog (Leijten and Van Waes, 2013), to take advantage of more extensive pause and revision analysis. Table 2 illustrates what the data looks like, exemplifying with one file from a DHH participant. The table displays two typical outputs: in the right column is found an extract from the so-called final text, i.e., the written product that the writer produced during the writing session. In the left column is the corresponding so-called linear text, i.e., a linear step-for-step representation of all the actions the writer engaged in while producing the text. The English translation in this example, as well as other text examples in the article, is deliberately as close to the Swedish original as possible, trying to “translate” the mistakes concerning spelling and morphology, including omissions of function words, that the participant makes.

**TABLE 2:** An example of the written product (to the left) and the corresponding writing process in the linear file (to the right).

Final text (Swedish original)	Linear text (Swedish original)
En snögig natt, där bor rosa pantern, han älskar sova. Det är bästa som han vet. Han sover jätte djupt. Men plötsligt hörde han någon knackar dörren. Han rusade ut och kikade om någon är där. Men han ser ingen.	<11.707> En <BACKSPACE3> <2.128> <BACKSPACE8> En snögig <BACKSPACE4> gig natten <2.888> <BACKSPACE3>, där bor ett hus <BACKSPACE9> r rosa pantern <BACKSPACE3>n <BACKSPACE2>r n <BACKSPACE2>m <BACKSPACE2>n <BACKSPACE1>, han älskar sova <15.642> <LEFT39> <RIGHT1> <LEFT1> <2.209> <RIGHT39> <2.078>. Han sover djupt och djupt. <BACKSPACE1> Men<2.337>plötsligt hörde han någon knackar <BACKSPACE1>de dörren<15.690>. Han rusar <BACKSPACE2>de u <BACKSPACE1> ut och kikade om någon är där. Men han såg inte att <BACKSPACE11>er ingen.
**Final text (English translation)**	**Linear text (English translation)**
A snowgy night, there lives the Pink Panther, he loves sleep. It’s best he knows. He sleeps very deeply. But suddenly he heard someone knocking the door. He rushed out and looked to see if anyone was there. But he sees no one.	[<11.707> A <BACKSPACE3> <2.128> <BACKSPACE8> A snowgy <BACKSPACE4> ogwy night <2.888> <BACKSPACE3>, there lives a house <BACKSPACE9> r Pink Panther <BACKSPACE3> n <BACKSPACE2> r n <BACKSPACE2> m <BACKSPACE2> n <BACKSPACE1>, he loves sleeping <15.642> <LEFT39> <RIGHT1> <LEFT1> <2.209> <RIGHT39> <2.078>. He sleeps deeply and deeply. <BACKSPACE1> But < 2.337>suddenly he heard someone knocki <BACKSPACE1> de the door <15.690>. He rushed out <BACKSPACE2> de u <BACKSPACE1> out and looked if someone is there. But she did not see that <BACKSPACE11> ees nobody.

The writer is a DHH participant. Pauses longer than 2 s are indicated with numbers between angular brackets, at the location where they occurred. Within angular brackets also indicate other actions, such as use of “backspace” or of arrow keys, like “left” or “right”. A notation like “<BACKSPACE3>” indicates three consecutive presses on the “backspace key”. In the English translation we have aimed to for a close mapping of the Swedish original, and we have also mirrored lexical, grammatical, and orthographic errors in the Swedish text.

### 2.4. Design and materials

The children were shown a two-paged cartoon about a Pink Panther, and asked to re-narrate it on the computer. They were informed that they could freely interpret the cartoon and use any name for the Pink Panther. They were also encouraged to use their own imagination. They were provided unlimited time to finish the story, with the primary reason to avoid time pressure, that in turn may result in uncompleted stories. Each child carried out the writing task without interruption, and their average total time on task was 32 min.

Before the writing session started, the children were informed that they would perform the writing task without assistance, but that one of the authors would sit slightly behind them during the whole test sessions. After the writing session, they were informed that their writing behavior had been recorded. The decision to reveal how the keystroke logging program worked after the writing was made to ensure that the writing performance would be as normal as possible, and to limit stress or pressure.

### 2.5. Procedure

The procedure was divided into three steps. First, possible participants were identified through networking, schools and through two CI-teams’ patient lists. Second, consent, and background questionnaire were distributed to those who approved to participate. The parents filled in the background questionnaire about their children’s linguistic, family, and school backgrounds and sent them back before the testing sessions started (through letters or via their children). The last and third step was the testing sessions. The data collection took place individually, in a quiet room in which the children started with the writing task and ended with the SignRepL2-test. Overall, the testing sessions, including instructions, writing task, and SignRepL2, lasted for around an hour.

### 2.6. Analyses of written products

For an overview and definition of the measures used for analyzing the written products, i.e., the final texts, we refer to Table 3. The written texts were transcribed and examined through a well-established computerized tools for corpus analysis, Computerized Language ANalysis (CLAN) (MacWhinney, 2000). CLAN was used for extracting the main part of the overall information on text length: number of words and word length; and of lexical measures: lexical density and lexical diversity. Microsoft Word was used for extracting information on number of words in the final text.

**TABLE 3:** The definitions of the measures used to examine the written products/final texts.

Measure	Definition
**Number of words**	A word is defined as a string of letters separated by spaces (or punctuation). The number of words were obtained through Microsoft Word’s in-build word count.
**Word length**	Word length is defined as the number of characters. The measure was calculated through using Computerized Language ANalysis (CLAN). The measure is an average length for all words in the text.
**Proportion of spelling errors**	The spelling errors in the final texts. Were identified and coded manually by one of the authors, and then extracted/calculated manually. To compare the proportion of spelling errors between participants, the number of spelling errors in the final texts were divided with the number of words in the final texts.
**Lexical diversity**	Measures of lexical diversity indicate the lexical variation in a text, i.e., the more unique words that are used, the higher the lexical diversity. In this study we used the measure “VocD” (Malvern et al., 2004), which is incorporated in the CLAN programs (MacWhinney, 2000). Automatically extracted by CLAN.
**Lexical density**	Measures of lexical density indicate how big proportion of the text that consist of lexical words (nouns, verbs, adjectives, and lexical adverbs). We use a measure of lexical density where the number of lexical words is divided by the total number of words (Halliday, 1985; Johansson, 2009). The distribution of lexical and function words was manually sorted by one of the authors, and CLAN was then used to generate the number of function words and lexical words, respectively.

We measure text length in number of words, following previous literature demonstrating that this is a stable measure of development in the age span (see Johansson, 2009). We have further included measures of lexical density and lexical diversity to describe the vocabulary development and lexical properties of the texts (see Johansson, 2009, for an overview). The measure of average word length is further included to give an indication on whether the length increases; since lexical words overall are longer than function words, this may mirror an increased use of lexical items. Finally, we have examined the proportion of spelling errors in the final text, since spelling difficulties has been reported to be a recurring issue for the DHH group, and for children in general in this age span.

### 2.7. Analyses of writing processes

This section describes the measures used for analyzing the writing processes, and we refer to Table 4 for a definition of each measure. Since the previous literature includes a variation in the measures for describing writing processes, we have made a selection of measures for our purpose of exploring the data. The selected measures originate from analyses provided by the keystroke logging programs ScriptLog and Inputlog, and are sometimes post-processed by the authors in Excel.

**TABLE 4:** The definitions of the measures used to examine the writing time, writing flow and pauses.

Analysis	Definition
**Writing time**	Writing time was defined as total time on task, i.e., the total writing time, in minutes, seconds, and milliseconds. This was automatically extracted by ScriptLog.
**Writing flow (offline)**	The offline writing flow was defined as the amount of text (measured in number of characters) the final text, divided by the writing time in seconds. The number of characters (i.e., letters, numbers, punctuation, and spaces) were automatically calculated by ScriptLog, and the offline writing flow was manually calculated by the authors.
**Writing flow (online)**	The online writing flow was defined as the amount of text (measured in number of characters) in the linear text, divided by the total writing time in seconds. The number of characters (i.e., letters, numbers, punctuation, and spaces) were automatically calculated by ScriptLog, and the online writing flow was manually calculated by the authors.
**Transition time**	The transition time between letters within a word has often been used as a measure of the writer’s typing proficiency, or transcription skills (Wengelin, 2006). We have used the median transition time (in seconds) between two consecutive keystrokes within a word, which is automatically extracted by ScriptLog.
**Pause percentage**	Pauses were defined as inactivity during typing. We used two pause criteria, to capture different processes: inactivity during 1 s or longer was used to examine low-level processes, such as transcription skills and spelling; inactivity during 4 s or longer was used to explore more high-level processes, connected to planning and revision. Pauses and pause time were automatically extracted by ScriptLog, and the pause percentage, or the proportion pause time of the total writing time, was obtained by the authors through manually dividing the pause time with the total writing time.
**P-bursts in characters (mean)**	A P-burst (or “pause burst”) is defined as a sequence of typed characters between pauses of a defined length, and is a measure that has been developed to indicate fluency during writing. This measure indicates how many written characters each burst contain, or in other words the number of characters that is produced before the next pause (of a defined length) occur. Different pause criteria can be set: for instance, would a criterion of 1 s render shorter bursts in number of characters than a criteria of 4 s. P-bursts in characters were automatically extracted by Inputlog.
**P-bursts in seconds (mean)**	A P-burst can also be measured in seconds, i.e., how many seconds of fluent writing that occur before it is interrupted by a pause (of a defined length). P-burst in seconds were automatically extracted by Inputlog.

An important purpose for studying writing processes in previous literature has been to capture fluency in text production (see Chenoweth and Hayes, 2001; Wengelin, 2006; Johansson, 2009), which is seen as an indication of how effortless a writer can produce her text. Fluency during writing will typically be calculated through a division of the number of linguistic units: words, or written characters (i.e., letters, punctuation) per time unit (seconds, minutes, or the whole time on task/total writing time). Fluency can further be related either to the linguistic units in the finally written text, that is, the written product (offline writing flow), or to the linguistic units that were produced during writing, and perhaps deleted during the writing processes (online writing flow). If the writer has deleted much text during writing, there will be a discrepancy between the two fluency measures. Fluency is thus connected to on the one hand pause time during writing (more and longer pause time will lead to a less fluent writing process), but also to revision and deleted characters (much deleted text, and many instances of revision will also lead to a less fluent writing process). To capture how the interplay of pauses and revision create fluency, the field of cognitive writing research has introduced several complex measures. Here we will use two measures of P-bursts, or “pause burst,” as a measure of either how many characters or how many seconds that a writer on average produces text without interruption of a pause. We equally use two measures of R-bursts, or “revision bursts,” which measures how many characters or how many seconds a writer on average produces text without interruption of a revision. The expectation is that more fluent writers in general are able to produce longer P-bursts and longer R-bursts.

Thus, to explore the writing processes and especially fluency, we have first included an overall measure of time on task or writing time. The measures of fluency include the writing flow offline (in number of produced characters in the final text divided by writing time), and writing flow online (in number of produced characters in the linear text divided by writing time), as well as the transition times, i.e., the writers’ typing speed between letters within a word, which is a measure that generally have been used to indicate transcription skills (Wengelin, 2006). The next cluster of measures concerns pausing, where we have included some of the measures automatically provided by Inputlog. The motivation for selecting these measures is to explore how automatization of transcription skills (as described by the model of Berninger and Swanson, 1994) and cognitive costs (following the capacity theory of writing described in McCutchen, 1996) will be shown in the data. We have further selected a measure related to pause location, that is pauses between sentences, following previous research (Spelman-Miller, 2006; Ailhaud et al., 2016) which have related the number of pauses and pause time between sentences to the writer’s planning behavior. For our pause analyses, we used a 1 s *ad hoc* criterion. Many previous writing studies use a 2 s criteria to exclude shorter pauses which may occur due to limited transcription skills (see Wengelin, 2006). However, since we are interested in exploring how the emerging transcription fluency relates to other writing processes and written products, we chose the 1 s criteria for this purpose, with the aim to explore the micro processes (related to such things as transcription skills and spelling).

Further, we have included some measures for examining revisions which can be shown in Table 5. This includes an overview of how many characters that were removed from, and inserted into, the texts, with the purpose to explore revisions related to more sophisticated use of lexicon. To further examine this, the revisions were coded *ad hoc* regarding whether they took place locally (i.e., in the close vicinity of the inscription point) or further away, which we here call global revisions. Revisions were also divided into smaller and bigger revisions in an attempt to capture a more mature revision behavior; previous research has demonstrated that when children develop their revision skills they engage in more deep revision, or semantic revision, involving lexical words (see Chanquoy, 2001, 2009). The difference between smaller and bigger revisions was operationalized into whether an instance of revision included the deletion of words of four or less characters (= smaller revision), or words of five or more characters (= bigger revision). From this *ad hoc* division we expected to roughly differentiate between deletions of function words (often 3–4 letters or less in Swedish, see Sigurd et al., 2004), and deletions of lexical words. Finally, we also looked at R-bursts, i.e., the number of characters or seconds between a revision event. This measure will say something about the fluency and accuracy of the writing, but also of how promptly the writer attends to possible errors in the texts.

**TABLE 5:** The definitions of the measures used to examine the revisions.

Analysis	Definition
**Removed characters in percentage**	The total number of removed characters were divided by the total number of produced characters. Automatically extracted by Inputlog
**Inserted characters in percentage**	The total number of inserted characters were divided by the total number of produced characters (in the linear text). Automatically extracted by Inputlog
**Bigger revisions (>5 characters)**	Bigger revisions were *ad hoc* defined as all revisions shorter than five characters were defined as a smaller revision, and all revisions longer than five characters were defined as a bigger revision. Automatically extracted by Inputlog, and then smaller and bigger revisions were separated through manual coding by one of the authors.
**Local or global revisions**	Revision location was for convenience *ad hoc* divided into two categories: local revisions, i.e., revisions occurring at the inscription point, or global revisions, i.e., revisions that were made further away from the inscription point, that is when the writer had used the mouse or arrow keys to move away from the inscription point to revise something. Automatically extracted by Inputlog.
**R-bursts in characters (mean)**	A R-burst (or “revision burst”) is defined as a sequence of consecutive typed characters produced between revisions (e.g., pressing backspace or delete). The measure is an attempt to illustrate fluency. The measure is automatically extracted by Inputlog.
**R-burst in seconds (mean)**	An R-burst in seconds is defined as the number of seconds of text production that occur between revisions. The measure is automatically extracted by Inputlog.

### 2.8. Description of statistical models

A correlation analysis was conducted between the written products measures and the writing process measures, and is found in Table 6. All the means and standard deviations (SD) on the participants’ written product measures as well as their writing process measures including pausing and revision can be found in Table 7, and the multiple regression analysis on these measures can be found in Table 8. See Supplementary material to Table 8 that can be found in the end of this paper. The statistical analyses were carried out through the program R (R Core Team, 2022).

**TABLE 6:** Correlation analysis.

	**Number of words**													
**Word length**		**Word length**												
**Spelling errors**		−0.37*	**Spelling errors**											
**Lexical diversity**	0.51**	0.45**		**Lexical diversity**										
**Lexical density**		0.578***			**Lexical density**									
**Writing time**			−0.33*			**Writing time**								
**Writing flow online**	0.37*		−0.46**				**Writing flow online**							
**Writing flow offline**	0.35*		−0.42**				0.97***	**Writing flow offline**						
**Transition time**	−0.36		0.56***				−0.83***	−0.77***	**Transition time**					
**P-bursts in characters**	0.41*									**P-bursts in characters**				
**Removed words in %**		0.32*			0.49**						**Removed words in %**			
**R-bursts in seconds**							−0.40*	−0.37*	0.44**	−0.63***		**R-bursts in seconds**		
**Bigger revisions**					0.35*						0.37*		**Bigger revisions**	
**Global revisions**														**Global revisions**

The table contains an overview of the measures (in bold). If a correlation was shown, the significance is indicated, otherwise the cell is empty.

*Significance at *p* ≤ 0.05 level, **significance at *p* ≤ 0.01 level, ***significance at *p* ≤ 0.001 level.

**TABLE 7:** DHH, CODA and hearing groups’ means and standard deviations (SD; in italics) for all measures that were investigated.

	DHH		CODA		Hearing	
	**M**	**SD**	**M**	**SD**	**M**	**SD**
**Written product**						
**Number of words**	305.7	(57.7)	434.1	*(269.6)*	269.9	*(109.0)*
**Number of characters**	1,646.8	*(363.9)*	2,275.4	*(1,388.2)*	1,528.5	*(591.2)*
**Word length**	4.2	*(0.2)*	4.1	*(0.2)*	4.0	*(0.2)*
**Proportion spelling errors**	2.4%	*(2.7)*	4.0%	*(3.5)*	3.2%	*(2.6)*
**Lexical diversity**	53.3	*(12.4)*	50.6	*(13.2)*	55.3	*(15.3)*
**Lexical density**	52.3	*(4.6)*	49.4	*(3.1)*	45.5	*(3.4)*
**Writing process**						
**Writing time in minutes**	32.0	*(12.3)*	32.1	*(13.1)*	33.0	*(15.6)*
**Number of characters (linear text)**	2,073.0	*(662.9)*	2,576.3	*(1,577.8)*	1,742.5	*(702.7)*
**Offline writing flow (characters/seconds)**	0.95	(0.36)	1.16	*(0.33)*	0.87	*(0.29)*
**Online writing flow (characters/seconds)**	1.18	(0.42)	1.31	*(0.33)*	0.98	*(0.40)*
**Transition time median**	0.36	*(0.17)*	0.29	*(0.09)*	0.38	*(0.16)*
**Pauses**						
**Pause percentage**	61.5%	*(8.7)*	60.2%	*(10.1)*	62.2%	*(10.7)*
**P-burst in number of characters**	7.5	*(3.5)*	9.3	*(3.8)*	6.2	*(3.4)*
**P-burst in seconds**	2.7	*(1.0)*	3.0	*(0.9)*	2.2	*(0.9)*
**Number of pauses between sentences**	26.9	*(19.8)*	23.2	*(12.9)*	28.6	*(23.1)*
**Pauses between sentences in seconds**	11.6	*(15.4)*	9.6	*(5.3)*	12.3	*(6.8)*
**Revision**						
**Removed characters in %**	18.6%	*(9.1)*	11.3%	*(3.4)*	11.6%	*(3.5)*
**Inserted characters in %**	4.8%	*(5.0)*	2.8%	*(2.9)*	2.8	*(3.1)*
**R-bursts in seconds**	16.1	*(11.9)*	14.9	*(4.6)*	19.0	*(5.4)*
**R-bursts in characters**	17.3	*(6.9)*	19.3	*(3.8)*	17.4	*(4.7)*
**Bigger revisions**	19.1%	*(10.6)*	12.8%	*(7.4)*	9.8%	*(7.7)*
**Global revisions**	9.5%	*(8.3)*	10.9%	*(6.8)*	11.4	*(9.1)*

**TABLE 8:** Multiple regression analysis.

	Model										
	Intercept		Age		Gender		Hearing		STS		Regression
Estimation/ standard errors	EST	SE	EST	SE	EST	SE	EST	SE	EST	SE	Bonferroni: *p* < 0.0125*
**Written product**											
**Number of words**	165.16	64.16	57.97	44.31	102.53	55.26	**152.3**	**65.65**	58.48	39.93	F(4, 31) = 2.797, *p* = 0.043*
**Number of characters**	844.8	334.1	314.1	228.4	**603.8**	**284.8**	**766.9**	**338.4**	323.9	205.8	F(4, 31) = 3.214, *p* = 0.026*
**Word length**	3.21	0.41	0.05	0.04	**0.17**	**0.05**	−0.02	0.06	0.06	0.03	F(4, 31) = 7.309, *p* < 0.0001***
**Proportion spelling errors**	0.26	0.09	−**0.02**	**0.01**	−0.00	0.01	0.00	0.01	0.01	0.01	F(4, 31) = 2.365, *p* = 0.075
**Lexical diversity**	44.45	5.15	**9.60**	**3.52**	**9.75**	**4.39**	3.15	5.22	−4.50	3.17	F(4, 31) = 3.278, *p* = 0.024*
**Lexical density**	0.47	0.12	−0.01	0.01	0.01	0.01	−0.03	0.02	**0.03**	**0.01**	F(4, 31) = 6.177, *p* < 0.0001***
**Writing process**											
**Writing time in minutes**	1,968.7	362.4	−103.3	308.9	227.0	247.7	73.1	367.0	−115.3	223.2	F(4, 31) = 0.240, *p* = 0.9134
**Number of characters (linear text)**	1,070.9	388.9	445.5	265.9	**765.5**	**331.6**	722.6	393.9	353.3	239.6	F(4, 31) = 3.593, *p* = 0.016*
**Offline writing flow (characters/seconds)**	0.747	0.139	**0.190**	**0.10**	0.060	0.118	**0.285**	**0.140**	0.116	0.085	F(4, 31) = 2.571, *p* = *0.05728*
**Online writing flow (characters/seconds)**	0.890	0.152	**0.251**	**0.104**	0.117	0.129	0.251	0.154	0.130	0.094	F(4, 31) = 3.51, *p* = 0.0178*
**Transition time median**	1.98	0.39	−**0.13**	**0.04**	0.00	0.05	−**0.13**	**0.05**	−0.04	0.03	F(4, 31) = 5.144, *p* = 0.0026**
**Pauses**											
**Pause percentage**	68.52	32.05	−0.86	2.95	2.76	3.69	−0.23	4.38	0.26	2.66	F(4, 31) = 0.1561, *p* = 0.9588
**P-burst in number of characters**	4.88	1.38	**2.78**	**0.94**	1.63	1.17	2.26	1.40	0.24	0.85	F(4, 31) = 3.486, *p* = 0.01835*
**P-burst in seconds**	2.02	0.38	**0.69**	**0.26**	0.43	0.33	0.39	0.38	0.07	0.24	F(4, 31) = 2.931, *p* = 0.0364*
**Number of pauses between sentences**	25.63	7.73	**15.1**	**5.29**	−1.55	6.59	2.96	7.83	−7.07	4.77	F(4, 31) = 1.515, *p* = 0.108
**Pauses between sentences in seconds**	56.17	32.12	−4.08	2.96	3.32	3.70	−2.75	4.39	0.24	2.67	F(4, 31) = 0.715, *p* = 0.5877
**Revision**											
**Removed characters in %**	0.15	0.02	0.02	0.02	0.03	0.02	−**0.06**	**0.02**	−0.00	0.02	F(4, 31) = 4.164, *p* = 0.008**
**Inserted characters in %**	0.02	0.01	**0.02**	**0.01**	0.02	0.01	−0.01	0.01	−0.01	0.01	F(4, 31) = 3.337, *p* = 0.02201*
**R-bursts in seconds**	106.07	21.50	−**7.68**	**1.98**	−2.47	2.47	−4.18	2.93	0.58	1.79	F(4, 31) = 4.639, *p* = 0.00473**
**R-bursts in characters**	44.86	16.65	−2.88	1.54	−0.20	1.92	0.53	2.28	1.71	1.38	F(4, 31) = 1.009, *p* = *0.4181*
**Major revisions**	0.17	0.04	0.02	0.03	0.01	0.03	−0.06	0.04	0.01	0.02	F(4, 31) = 1.953, *p* = 0.1265
**Global revisions**	0.01	0.27	0.01	0.02	0.01	0.03	0.01	0.04	−0.02	0.02	F(4, 31) = 0.264, *p* = 0.8988

The table shows a model with four predictors: Age, gender, hearing and STS.

*The significance level at *p* ≤ 0.05 level, **at *p* ≤ 0.01 level, and ***at *p* ≤ 0.001 level were all colored in gray.

Significant measures are indicated in bold. Effects in shaded cells are significant with *p* < 0.0125. However, the Bonferroni corrected significant levels are marked in dark gray. The Bonferroni level in the model is 0.05/4 = 0.0125*.

## 3. Results

In this section the results are presented, starting with a correlation analysis which explores the overall relationships between some of the overarching writing process and written product measures, see Table 6. Next, we present Table 7 that shows the means and standard deviations of all the measures, collapsed over the three groups: DHH, CODA, and the hearing group. Finally, we report the results from a multiple regression analysis to demonstrate which measures that were predicted by age, gender, hearing, and STS, see Table 8.

### 3.1. Summary of the results of the correlation analysis

In the following, we report the significant correlations revealed in the correlation analysis. The analysis serves the purpose of identifying general trends in how properties of the written texts and the writing processes are related, independent of the writers’ background.

First, a significant positive correlation was found between text length measured as number of words and lexical diversity. In addition, the number of words correlated positively with both writing flow offline and online, and P-bursts in characters. Finally, number of words correlated negatively with transition times, in other words: writers with shorter transition times wrote longer texts. Word length correlated positively with lexical diversity and lexical density. There was further a negative correlation with spelling errors. In other words, writers with many longer words (i.e., an indication of many lexical words) had fewer spelling errors. Spelling errors correlated positively with transition times, meaning that writers with longer transition time (i.e., an indication of faster typists) also had more spelling errors. In addition, spelling errors were negatively correlated with writing time and writing flow offline and online. All of this indicates that writers with many spelling errors also wrote less fluently, but in a shorter time. Lexical density correlated positively with bigger revisions (i.e., removing five or more characters during one instance of revision). In all, this shows that the proportion content words increases when longer revisions were made.

Writing flow online and offline correlated with each other. In addition, both measures correlated negatively with transition time and R-bursts in seconds. In other words, writers with higher fluency online and offline had shorter transition times and shorter R-bursts, i.e., had less time between their revisions. Transition time correlated positively with R-bursts in seconds, indicating that writers with longer transition times also had longer time between their revisions. P-bursts in characters, correlated negatively with R-bursts in seconds, indicating that writers who wrote many characters between pauses, also had shorter time between their revisions. In sum, writers here demonstrated lower fluency as measured through the frequency of revision, but comparably high fluency as measured by the amount of text produced between pauses.

### 3.2. Summary of the results of the written product measures

Bonferroni correction was used as an adjustment method for multiple comparisons. The critical *p*-value 0.05 was divided by four (the number of predictors) which means that the Bonferroni correction critical *p*-value is 0.0125*. Following our explorative objective to identify areas where the DHH group may face challenges due to their linguistic background, we report (and later discuss) the significant differences (both with and without Bonferroni corrections).

Number of words was predicted by hearing which means that the hearing writers produced more words than their DHH peers. Number of characters (final text) was predicted by hearing and gender which means that the girls and hearing writers produced more characters than boys and DHH peers. Word length was, through Bonferroni correction, predicted by gender, which means that the average word length was longer in the girls’ texts. Proportion of spelling errors was predicted by age which means that the older writers had a smaller proportion spelling error. Lexical diversity was predicted by age and gender which means that older writers and girls had higher lexical diversity than younger writers and boys. Lexical density was predicted by STS, which means that writers with higher knowledge of STS had higher lexical density.

### 3.3. Summary of the results of the writing process measures

Number of characters (linear text) was predicted by gender which means that girls produced more characters than boys. Writing flow (offline) was predicted by hearing and age which means that the older writers and the hearing writers produced more text in shorter time than younger writers and the DHH group. Writing flow (online) was predicted by age which again means that the older writers produced more text in shorter time, also when the writing process was examined. Transition time was, through Bonferroni correction, predicted by hearing and age which means that hearing and older writers had a faster transition time than the DHH group and younger writers.

P-bursts in number of characters was significantly predicted by age which means that older writers had longer P-bursts in characters than younger. P-bursts in seconds was significantly predicted by age which means that older writers had longer P-bursts than younger. Number of pauses between sentences was predicted by age which means that older writers to a greater extent paused between sentences than younger.

Percentage removed characters was, through Bonferroni correction, predicted by hearing which means that DHH group removed more characters than their hearing peers. Percentage inserted characters was predicted by age which means that older writers inserted more characters than younger. R-bursts in seconds was, through Bonferroni correction, predicted by age which means that older writers had shorter R-bursts than younger.

### 3.4. Illustrating examples

In this section, we supplement the quantitative findings above with graphic illustrations of a selection of the measures, in order to visualize some of the findings from the previous analyses, and to understand possible differences between the groups.

**FIGURE 1:**
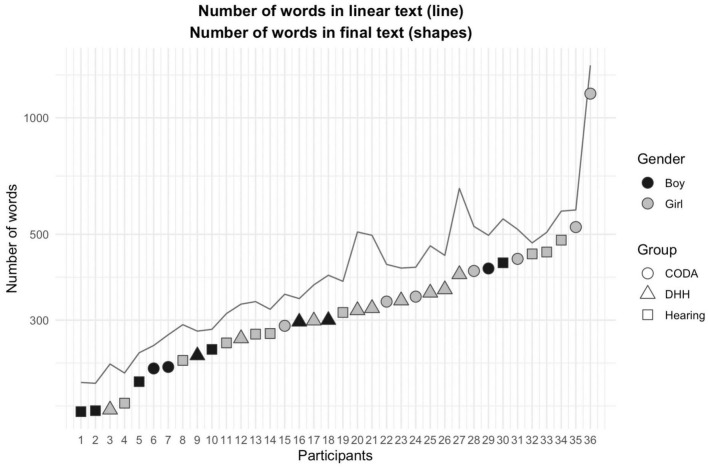
The number of words in final and linear texts for the three groups. The figure illustrates each of the children’s number of written words in the linear and final texts. The black and gray shapes in the lower part of the graph shows the individual children’s number of words in the final text. The upper gray line illustrates each of the corresponding number of words the in the linear text (that is, how many words that were produced during the whole writing session). The distance between the shapes and the line is thus an illustration of the number of words that were removed during the writing of the texts. The gender and the groups are also indicated: the boys are plotted in black, and the girls are plotted in gray. Children in the DHH group are plotted with a triangle, the CODA children with a circle, and the hearing children with a square.

Figure 1 displays the individual text length for each child, showing both the number of words in the written product/the final text (the lower line of squares, triangles and circles) and the number of words produced in the linear text (in the upper line). The texts are ordered from the shortest to the longest. As is demonstrated, children in the DHH, CODA, and hearing groups are distributed all across the figure. However, one can note that the hearing children in our sample write the longest texts. Also, the boys are in minority in the sample, and their texts are mostly found among the shortest 50% of the texts.

**FIGURE 2:**
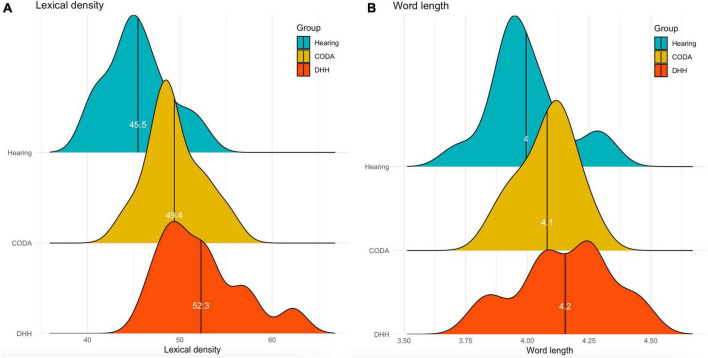
**(A,B)** The distribution of lexical density and average word length (in letters) divided over groups: the hearing group in blue, the CODA group in yellow, and the DHH group in red.

Figure 2A shows the distribution of lexical density (i.e., the proportion of lexical words in the texts), indicating that the DHH group had a bigger variation between participants. The same pattern is found for the average word length, shown by Figure 2B, where the DHH group displays more variation in word length than the other groups.

**FIGURE 3:**
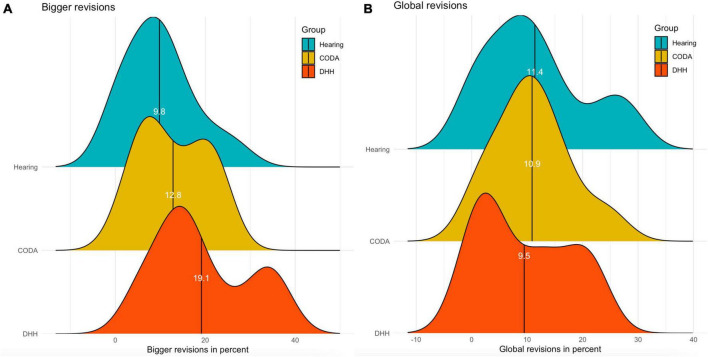
**(A,B)** The distribution of bigger and global revisions divided over groups: the hearing group in blue, the CODA group in yellow, and the DHH group in red.

Figures 3A, B show two revision measures: how the groups engaged in bigger revisions (i.e., deleting words longer than five characters) and in global revisions (i.e., deleting one or more characters but not at the inscription point). This illustrates that the distribution between participants for the bigger revisions is more spread out for the DHH group, and that regarding the global revisions, the three groups demonstrate a more similar distribution.

## 4. Discussion

In this section we discuss the research questions in relation to both correlation and multiple regression analyses. As we used Bonferroni correction as an adjustment method for multiple comparisons, we will in particular emphasize which variables had a *p*-value lower than 0.0125 because they are the study’s strongest predictors. However, given the study’s explorative attempt, we will also discuss the significant measures (*p* < 0.05*) that were not Bonferroni corrected. We motivate this by our overall purpose to identify such factors that may be interesting to address in future studies.

### 4.1. The relation between written products and writing processes

We begin by addressing the first research question, that is how written products and writing processes are realized and related in narrative writing for children age 10 to 12. The overall results indicate that longer texts are associated with a more varied lexicon, which confirms previous descriptions of linguistic development where text length and vocabulary growth are connected and interpreted as indications of matureness (Scott, 1988; Crossley et al., 2014).

The longer texts are further connected with more fluency during writing. This relates both to faster transition times between letters within words—a measure that often has been used to categorize the writers’ automatization of transcription skills—and online and offline writing flow, i.e., production measures of how many characters in the final as well as the linear texts that were produced during a given time. Another fluency measure connected to longer texts was P-bursts (i.e., how many characters that were typed between pauses longer than 1 s) that were associated with more fluent writing. This is all in line with theories about automatization which suggest that when transcription skills are established, this will decrease the cognitive costs on low-level processes for the writers, and allow more elaborations on lexicon and expansion of the content and events in the texts (Berninger and Swanson, 1994; McCutchen, 1996; Fayol, 1999). Increased fluency was also associated with proportion spelling errors, indicating that less skilled typists also were occupied with attending to orthography.

A further observation is that lexical diversity (i.e., the variation of words), and lexical density (i.e., the proportion of lexical words) are not correlated. However, both measures correlate with the average word length, in those texts with longer average word length have higher lexical density and diversity. This is logical, since many function words are short (articles, pronouns, conjunctions, prepositions), and texts with many function words will per definition have lower lexical density. They also tend to have less variation, since most of the linguistic variation in Swedish—just as in English and many other languages—will occur due to additions of lexical words, which overall are longer. The lexical properties of the texts are also correlated with revision behavior, for instance writers with increased lexical density seem to erase longer strings of characters (what we here called bigger revisions, consisting of five letters or more). This can perhaps be interpreted that writers who engaged in bigger revisions to a higher extent produced texts that were more lexically sophisticated. It should also be noted that the children, independent of background, demonstrated a similar engagement in global and bigger revisions. In sum, the lexical aspects and the relation to revision behavior seem complicated and calls for further attention in future studies.

Similar to von Koss Torkildsen et al.’s (2016) study on Norwegian 8-year-old children (thus slightly younger than in our sample) we found that the more fluent writers (as measured by increased transition times, and longer P-bursts) also revised more. Interestingly, writers who produced more characters between short pauses (in this case indicated by P-bursts of 1 s) had shorter time between their revisions. This finding may be counterintuitive—that is, that one may think that writers with a profile with lower fluency may revise more. The study by von Koss Torkildsen et al. (2016) showed that children with higher reading and spelling skills made the largest number of revisions, and also that the number of revisions predicted the length of the texts. One explanation that is proposed is that although children become faster typists, they are in the beginning still not so accurate, and this causes typing mistakes that need correction.

To illustrate what the writing process can look like for a writer who is fluent, but revise a lot, we have included an example in Table 9, from a text written by a girl in the CODA group. The example shows all pauses longer than 1 s. The linear file clearly demonstrates that the writer quite fluently produces rather long strings of characters between the pauses, i.e., a sign of long P-bursts. The examination of revision through R-bursts shows that the writing is frequently interrupted by use of backspace, where the writer erases one or more characters. These revisions typically can be associated with spelling errors or writing mistakes which have occurred due to pressing the wrong key; these mistakes are apparently quickly detected and attended to. Whether the writer recognizes the mistakes through haptic input, or visual (or both) is difficult to say, but given the rapid corrections we suggest that the occurrence of this type of revisions is not attributed to lack of orthographic knowledge.

**TABLE 9:** A text example of a final text and corresponding linear text by a CODA writer.

CODA writer: final text (Swedish original)	CODA writer: linear text (Swedish original)
Rufus ville bara ut från drömmen och in i verkligheten. Han märkte att tassavtrycken ledde [honom] tillbaka till sängen.	Rud <BACKSPACE1> fus il <BACKSPACE2> ville bara ut från drömmen<1.379>och in i veklighwte <BACKSPACE4> <1.047>heten. <11.998>Han m <1.075>ärkte att tass <BACKSPACE1> avtryckrn <BACKSPACE3> <1.021> en lede to <BACKSPACE1> illbaka till sänen <BACKSPACE2> <1.213> <BACKSPACE1> g <BACKSPACE1> <1.422> gen
**CODA writer: final text (English translation)**	**CODA writer: linear text (English translation)**
Rufus just wanted to get out from the dream and get inside to the reality. He noted that the paw prints led [him] back to the bed	Rud <BACKSPACE1> fus an <BACKSPACE2> just wanted get out from the dream<1.379>and get inside the realiwty <BACKSPACE4> <1.047> lity. <11.998>He n<1.075>oted that the paw <BACKSPACE1> priyt <BACKSPACE3> <1.021>nts led bs <BACKSPACE1> ack to the bd <BACKSPACE2> <1.213> <BACKSPACE1> e <BACKSPACE1> <1.422>ed

The example illustrates the revision procedure, e.g., the length of strings of characters and words produced between revisions, or what in other words is called R-bursts. Texts within square brackets in the final text indicate additions we have made in the original text, to facilitate the reading and understanding of the text.

To sum up, the overall picture is that for the writers in our study expansion of text length, vocabulary growth, increase in writing fluency and engagement in revisions are highly connected, just as previous studies have described as characteristic of more mature writing in this age span.

### 4.2. Background factors and prediction of written product measures

After having addressed the overall correlation between written products and writing processes in the previous section, we now turn our interest to the second research question that deals with how the background factors age, gender, hearing, and STS can affect the written products. In other words, we outline what factors that predicted text length and lexical properties in the final texts. We here discuss all results that our model found significant.

From the results we learn that age predicted the proportion of spelling errors and lexical diversity. In other words, the texts by the older children had a lower proportion spelling errors which may be a reflection of better orthographic knowledge. Similarly, texts with higher lexical diversity can be a sign of a more varied lexicon—both are factors that previously have been associated with more mature texts (Fayol, 1999; Johansson, 2009; Löhndorf, 2021; Gärdenfors, 2023). In addition, gender predicted the number of characters in the final texts, average word length, and lexical diversity. The means show that it is the girls who outperform the boys, and thus write longer texts, with more varied lexicon and longer words—again all features typically associated with more advanced writing skills. Gender differences were expected, since it has repeatedly been shown in previous studies on writing in this age group (Kanaris, 1999; Zhang et al., 2019; Truckenmiller et al., 2021; Gärdenfors, 2023). Since our population has a higher proportion of girls among the older children, we cannot exclude that this is causing the gender effect.

Further, hearing predicted text length in both number of words and number of characters in the final texts, which suggests that the writers with a hearing background (the CODA group and the hearing controls) had some advantages that enabled them to write longer texts. One advantage is perhaps that more processes have been automatized.

Finally, knowledge of sign language predicted a higher lexical density. Lexical density is a measure of the percentage of lexical words in the text, or, in other words, how big the proportion is of nouns, verbs, adjectives, and lexical adverbs. Since lexical density is a percentage measure, fewer function words (like articles, pronouns, prepositions, and conjunctions) will increase the lexical density. DHH children’s texts have been associated with higher lexical density in previous studies (e.g., Singleton et al., 2004; Asker-Árnason et al., 2012; Gärdenfors, 2021), but the question remains how this finding should be interpreted. In the literature describing linguistic properties in spoken and written texts (e.g., Halliday, 1985), higher lexical density is connected to more written-like texts, and in the developmental literature of children with L1 background, it is associated with more mature writers (see Berman and Verhoeven, 2002; Johansson, 2009). However, the situation may be different for the DHH group, and Asker-Árnason et al. (2012) have suggested that for this group, a higher lexical density may instead signal a less-developed grammar, where compulsory function words are missing. If we look at the text example in Table 2, we can understand why such an interpretation seems legitimate. Table 10 contains the same final text, from a DHH writer, as in Table 2, but this time we have added necessary function words (in bold) that were omitted by the writer (see the uppermost text). For comparison, we also include a text by one writer in the CODA group (see the bottom text), which—as is visible—does not lack necessary function words. In both texts we have indicated the lexical words (nouns, verbs, adjectives) in italics. Thus, both texts contain many content words, but the text by the DHH writer contains less function words.

**TABLE 10:** Two text examples of final texts from a DHH writer (the first text), and a CODA writer (the second text).

DHH writer: final text (Swedish original)	DHH writer: final text (English translation)
En *snögig natt*, där *bor rosa pantern*, han *älskar* **att** *sova*. Det *är* **det** *bästa* som han *vet*. Han *sover jätte djupt*. Men *plötsligt hörde* han **att** någon *knackar* **på** *dörren.*	A *snowgy night*, there *lives* the *pink panther*, he *loves* **to** *sleep*. It’s **the** *best* he *knows*. He *sleeps very deeply*. But *suddenly* he *heard* **that** someone *knocking* **at** the *door*.
**CODA writer: final text (Swedish original)**	**CODA writer: final text (English translation)**
När *rosa katten gick* ut med *säcken* så *skärde musen* ett *hål* i *säcken* och *hoppade* ut. *Rosa katten sparkade* ut *säcken* utan *musen*. Sen *hörde rosa katten snarkande* från *sängen*. *Rosa katten la* ut en *råttfälla*.	When the *pink cat went* out with the *sack*, the *mouse cutted* a *hole* in the *sack* and *jumped* out. The *pink cat kicked* the *sack* out without the *mouse*. Then the *pink cat heard snoring* from the *bed*. The *pink cat laid* a *mousetrap*.

The examples illustrate the proportion lexical words in the texts. The words in italics indicate lexical words. The bold words in the first text indicate necessary function words that were omitted in the original text (cf. Table 2).

Previous studies have put forward two main explanations for overall higher lexical density by the DHH group. The first explanation is that it occurs due to influence—or transfer—from the knowledge of sign language, which is generally described as a language with an abundance of descriptive words (Asker-Árnason et al., 2012). The second explanation is that the DHH group’s hearing loss may result in that that they to a limited extent can rely on influence, or transfer, from spoken language, and that this leads to higher lexical density. This explanation builds on the fact that a limited auditory input (quantitatively and qualitatively) may lead to that the DHH group—in spite of using hearing technology—do not always pick up on function words, and that this will especially affect unstressed articles and particles (Gärdenfors, 2021, 2023). The findings from the present study where we compare DHH and CODA writers—two groups with knowledge of sign language, but where the CODA writers are hearing, and do show any pattern of omitted function words in their texts—give support to the latter explanation.

Thus, it seems as if we are dealing with two effects at the same time: on the one hand, the CODA group generally writes longer text (see Figure 1), which for this age is associated with better writing skills. In addition, this group to a high extent has higher lexical density, which generally is attributed to texts that are more written-like and mature. On the other hand, the DHH group to some extent omits function words, which leads to texts with higher lexical density in the quantitative analysis. The omission must, however, not be seen a sign of maturation, but instead of more ungrammatical texts (following the argument by Asker-Árnason et al., 2012). As is shown in Figure 2A, it may only be a few children in the DHH group that have high lexical density, and at this point we do not know if these are children who omit more function words, or children who have more advanced, written-like texts. However, one overall conclusion is that quantitative approaches for describing lexical properties in written texts may hide more complicated patterns.

### 4.3. Background factors and prediction of writing process measures

In this section we discuss the third research question and the findings related to how our background factors age, gender, hearing, and STS can affect the writing processes. Also here, we discuss all the results that our model found significant.

The results revealed that age predicted online and offline writing flow and faster transition times between letters within words. Age was also a predictor of longer P-bursts in characters and longer P-bursts in seconds. P-bursts in characters indicated how many characters that were written between pauses of 1 s, and P-bursts in seconds how many seconds of fluent writing that elapsed between pauses of 1 s or longer, and the older writers thus wrote more characters and were generally able to writer during a longer time before they needed to pause. Age also predicted shorter R-bursts, thus indicating that the older writers wrote less characters before they engaged in revising (mostly deleting the just written characters). Taken together, the examination of P-bursts and R-bursts characterizes older writers with more fluent production of texts, but the frequently occurring revisions indicate less accuracy in typing. Finally, age predicted more pauses between sentences, while gender did not predict any of the writing process measures.

In conclusion, age was associated with several measures that points to higher writing fluency. Given the developmental model of Berninger and Swanson (1994) this can be expected. The children in our study are exactly in the age span (grade 4–6) which is described to have established basic transcription skills (including typing and spelling) and are expanding their writing to be more engaged in high-level processes such as revision. The finding that age predicted more pauses between sentences may indicate a higher degree of planning—supported by previous findings (e.g., Ailhaud et al., 2016) who attribute pauses in clause boundaries to planning.

Hearing predicted transition time, writing flow online and offline. In other words, children with DHH background demonstrated lower fluency, which is in line with previous findings by Asker-Árnason et al. (2012). But there was also a negative prediction in regard to percentage removed characters—in other words, the DHH writers deleted more. The knowledge of sign language did not predict any writing process measures.

In Table 11 below, we illustrate the type of revisions that can occur during writing for the DHH group. The table shows part of a final text and a corresponding linear text. The linear file is a bit difficult to decipher, but it shows that the first sentence *En natt så var det en stormig natt* (“One night it was a stormy night”) was written with a constant deletion of words; the writer tries using “cold” and “snowy,” rejects these lexical choices and eventually rewrites the introduction several times. This may be an illustration also of the higher percentage of bigger deletions found for this group (see also Figure 3A). We can further note that several deletions occur due to spelling mistakes: for instance, *kolla* (“check” in the erroneous infinitive form) instead of *kollade*; *vämn* (“left”) instead of *vänster*. Note, that the writer does not particularly engage in changing or removing function words, which hints to the fact that the omission of function words is present from the first draft and remains constant.

**TABLE 11:** A text examples illustrating the final and linear text by a DHH writer.

Final text (Swedish original)	Linear text (Swedish original)
En natt så var det en stormig natt. Rosa Panthern skulle sova med sin värme kudde. Han hörde knack ljud från dörren: Knack Knack. Han gick ut och kolla på höger och vänster holl men ingen var där!?	<1.751> <1.061> Det var kalt stormig <5.324> snö <BACKSPACE16> snö och <BACKSPACE16> <2.816> R <BACKSPACE1> En natt so <BACKSPACE1> å var det kalt <BACKSPACE6> en stormig natt <19.511> <BACKSPACE1> <5.042> <1.876> <2.202>. h <BACKSPACE1> Han gick ut och kolla <2.221> och udrade <BACKSPACE7> <2.434> kollade <2.318> <BACKSPACE8> vr <BACKSPA CE2> <5.217> virde hu<1.348> <BACKSPACE8> <3.922> <BACKSPACE5> på hÖ <BACKSPACE2> j <BACKSPACE1> höger och vämn <BACKSPACE2> nster <2.380> holl men ingen var där <4.468> <BACKSPACE1> <2.975> ! <1.170> ? <4.346> <BACKSPACE1> ä <BACKSPACE2> <1.557> hå <2.940> <BACKSPACE1> o <1.920> <BACKSPACE2> å <2.072> <BACKSPACE1> oll <BACKSPACE1>
**Final text (English translation)**	**Linear text (English translation)**
One night it was stormy night. The Pink Panther would sleep with his heating pad. He heard knocking sounds from the door: Knock Knock. He went outside and look on right and left hollds but no one was there!?	<1.751> <1.061>It was colld stormy <5.324> snow <BACKSPACE16> snow and <BACKSPACE16> <2.816> P <BACKSPACE1> One night sou <BACKSPACE1> o was it cold <BACKSPACE6> a stormy night <19.511> <BACKSPACE1> <5.042> <1.876> <2.202>. h <BACKSPACE1> He went out and check <2.221> and wodered <BACKSPACE7> <2.434> checking <2.318> <BACKSPACE8> by <BACKSPACE2> <5.217> by rI <1.348> h <BACKSPACE8> <3.922> <BACKSPACE5> on rI <BACKSPACE2> j <BACKSPACE1> right and left <BACKSPACE2> ft <2.380> sade but no one was there <4.468> <BACKSPACE1> <2.975> ! <1.170> ? <4.346> <BACKSPACE1> ä <BACKSPACE2> <1.557> hå <2.940> <BACKSPACE1> o <1.920> <BACKSPACE2> å <2.072> <BACKSPACE1> old <BACKSPACE1>

The linear file shows the high amount of revisions, where words are replaced with synonyms.

Overall, the explorations of the writing processes propose that the DHH group is occupied with changes at the word level, both regarding lexical choice and spelling. The engagement in changes at the word level may partly be explained as a trend common for the age group— Johansson (2000) reported that hearing 10-year-olds were engaged in deletions of words—and a supplementary explanation is that the DHH group is aware of their linguistics limitations during writing and compensates for this by more revision. The consequence is a “here and now focus” (cf. the knowledge teller, described by Bereiter and Scardamalia, 1987), where the most recent text will be repeatedly revised—mostly with success (!); the final texts have few spelling errors and are lexically diverse and dense. But following the models of working memory capacity (Just and Carpenter, 1992) and the capacity theory of writing (McCutchen, 1996) this behavior will be cognitively taxing, and leave little room for engaging in more high-level processes, or address planning issues on a discourse level. To conclude, future studies—using qualitative as well as quantitative approaches—to address issues regarding how and why lexical choices are made, and how lexical measures relate to syntactic development and rhetoric skills would be very fruitful to conduct. This seems especially important, given that quantitative methods of exploring lexical properties of texts may hide significant differences to why a certain phenomenon—like high lexical diversity—occur.

## 5. Conclusion

Here, we return to the overall question of how product and process are connected. The explorative nature of the present study has identified several areas that calls for further research. One such factor is how strong age is as a predictor for the outcome of the properties of the final texts and the writing processes. The establishment of basic transcription skills proves to be a key issue—something which also is stressed in Berninger and Swanson’s model of writing development: when the basic transcription skills are developed, this will free up cognitive capacity for engaging in more demanding tasks, such as producing longer texts and use a more varied lexicon. In our results, the correlation analysis, as well as the predictor age, support this. The older writers among our participants demonstrate higher fluency, and more mature texts, expressed in text length and lexical properties. The relation can have several explanations, where one is that when a writer adds more events to a story, this will on the one hand increase the text length, and on the other hand will adding new information (perhaps through expansions in the form of descriptions and explanations) result in more variation in lexicon.

That age is a very strong predictor for the writing outcome is important to take into consideration in the design of future studies on DHH population, since variation in writing proficiency due to age may hide other findings when the age range is big. But since the DHH population is small, it is challenging to keep the age range within one study small, while still controlling for other factors, which also is shown by the relatively big age range in previous studies of this population (e.g., Asker-Árnason et al., 2012; Arfé, 2015; Breland et al., 2022; Gärdenfors, 2023).

Earlier studies have argued that writing seems to be more challenging for the DHH group, compared to the children with hearing background (e.g., Arfé, 2015; Mayer et al., 2016; Mayer and Trezek, 2018; Breland et al., 2022). Our data support the idea that knowledge of spoken language and of sign language may influence the group’s writing performance. We have seen that the DHH group compares with hearing peers in many ways, both regarding the written product and the writing processes. However, some findings stand out. One is the high lexical density, which can be attributed to the lack of necessary function words in the final texts, something we explain by limitations in the auditory input—qualitatively as well as quantitatively (see Brännström et al., 2022). Particularly unstressed function words (e.g., articles, prepositions and pronouns) seem to go unnoticed by the DHH group. We propose that future research direct more attention to qualitative studies of how function words are acquired and used by the DHH group.

We further explored the importance of STS knowledge for writing performance. Our findings suggest that knowledge of STS predicted an increased lexical density. We discuss these findings above, suggesting that there may be two coinciding phenomena causing this effect—on the one hand that the CODA writers demonstrate a generally more mature writing behavior, in using a higher proportion of lexical words, and on the other hand that the DHH writers omit some function words which also leads to higher lexical density. To understand how and if knowledge of STS affects the DHH group’s writing another study design is needed. Future studies can for instance compare two DHH groups (within as small age range as possible), with and without STS knowledge.

One implication of the present study is the kind of support and instructions the DHH group requires in an educational setting. This is particularly important when DHH children attend mainstream classes, where the teachers meet children with reading and writing difficulties originating from different causes (e.g., dyslexia, limited linguistic input, second language background). An increased awareness is needed regarding the specific challenges the DHH group faces in a classroom. Examples include to pay particular attention to grammar issues that are normally not in focus for hearing children, who can use their linguistic spoken skills as a fundament for lexical choices and grammar in writing, and for whom omissions of function words are rare. Since the DHH children’s cognitive attention—at least in this age span—foremost lies on the word level, they will also need support for developing writing strategies that expand their focus to other linguistic levels—such as composing and revising. As an example, Strassman et al. (2019) (see also Wolbers et al., 2022) suggest using the SIWI model (Strategic and interactive writing instructions) for this purpose, where children and teachers explicitly participating in shared writing activities, and where the teachers highlight the use of such writing processes as planning, reading, and revising.

## Data availability statement

The raw data supporting the conclusions of this article will be made available by the authors, without undue reservation.

## Ethics statement

The studies involving human participants were reviewed and approved by the Etikprövningsnämnden in Stockholm. Written informed consent to participate in this study was provided by the participants’ legal guardian/next of kin.

## Author contributions

MG was mainly responsible for the data collection, analysis, and dissemination of the results and was involved in writing parts of the manuscript. VJ was the head main writer of the manuscript. Both authors contributed conceptually to everything in this manuscript.
