# Supplementary material for: Written products and writing processes in Swedish deaf and hard of hearing children: an explorative study on the impact of linguistic background
Source: Front Psychol. 2023 May 9;14:1112263. doi: 10.3389/fpsyg.2023.1112263 (PMC10203585; doi:10.3389/fpsyg.2023.1112263)
Supplement: Supplementary file 1 [file Table_1.DOCX]

|  | | **MODEL 1 (with gender)** | | | | | | | | | | | | | | | **MODEL 2 (without gender)** | | | | | | | | | | | | | | | | | |  |  |
| --- | --- | --- | --- | --- | --- | --- | --- | --- | --- | --- | --- | --- | --- | --- | --- | --- | --- | --- | --- | --- | --- | --- | --- | --- | --- | --- | --- | --- | --- | --- | --- | --- | --- | --- | --- | --- |
|  | | **Intercept** | | **Age** | | **Gender** | | **Hearing** | | **STS** | | | | | | **Regression** | **Intercept** | | | | | | **Age** | | | **Hearing** | | **STS** | | | | | **Regression** | |  |  |
|  | **Estimation/**  **standard errors** | EST | SE | EST | SE | EST | SE | EST | SE | EST | SE | | | | | **Bonferroni:  *p* <** **0.002**** | EST | | | SE | | | EST | | SE | EST | SE | EST | | | | SE | **Bonferroni:   *p* <** **0.002**** |  |  |  |
| **WRITTEN PRODUCT** | **Number of**  **words** | *165.16* | 64.16 | 57.97 | 44.31 | 102.53 | 55.26 | **152.3** | **65.65** | 58.48 | 39.93 | | | | | F(4,31) = 2.797,  *p* = .043* | *240.52* | | | 52.41 | | | 62.24 | | 45.91 | **146.14** | **68.02** | 57.82 | | | 41.43 | | F(3.32)= 2.399, *p =* .0862 |  |  |  |
|  | **Number of**  **characters** | *844.8* | 334.1 | 314.1 | 228.4 | **603.8** | **284.8** | **766.9** | **338.4** | 323.9 | 205.8 | | | | | F(4,31) = 3.214,  *p* = .026* | *1288.6* | | | 274.2 | | | 339.2 | | 240.2 | **730.2** | **355.9** | 320.0 | | | 216.8 | | F(3.32)=2.513, *p* = .07611 |  |  |  |
|  | **Word length** | *3.21* | 0.41 | 0.05 | 0.04 | **0.17** | **0.05** | -0.02 | 0.06 | 0.06 | 0.03 | | | | | F(4,31) = 7.309,  *p <* **.0001***** | *4.09* | | | 0.050 | | | 0.058 | | 0.045 | -0.030 | 0.065 | 0.055 | | | 0.04 | | F(3.32) = 3.765, *p* = .0217* |  |  |  |
|  | **Clauses** |  |  |  |  |  |  |  |  |  |  |  |  |  |  |  |  |  |  |  |  |  |  |  |  |  |  |  |  |  |  |  |  |  |  |  |
|  | **T-units** | *24.96* | 6.18 | 3.90 | 4.22 | **11.53** | **5.27** | 7.10 | 6.30 | 4.24 | 3.81 | | | | | F(4,31) = 2.167,  *p* = .096 | *33.43* | | | 5.095 | | | 4.383 | | 4.463 | 6.387 | 6.612 | 4.165 | | | 4.027 | | F(3.32) = 1.157,  *p* = .03412* |  |  |  |
|  | **Proportion**  **Spelling errors** | *0.26* | 0.09 | **-0.02** | **0.01** | -0.00 | 0.01 | 0.00 | 0.01 | 0.01 | 0.01 | | | | | F(4,31) = 2.365, *p* = .075 | *0.0291* | | | 0.009 | | | **-0.02** | | **0.008** | 0.004 | 0.012 | 0.008 | | | 0.007 | | F(3.32) = 3.152,  *p = .03825** |  |  |  |
|  | **Lexical**  **diversity** | *44.45* | 5.15 | **9.60** | **3.52** | **9.75** | **4.39** | 3.15 | 5.22 | -4.50 | 3.17 | | | | | F(4,31) = 3.278, *p* = .024* | *51.62* | | | 4.25 | | | **10.00** | | **3.73** | 2.56 | 5.521 | -4.56 | | | 3.362 | | F(3.32)=2.428, *p = .08349* |  |  |  |
|  | **Lexical**  **density** | *0.47* | 0.12 | -0.01 | 0.01 | 0.01 | 0.01 | -0.03 | 0.02 | **0.03** | **0.01** | | | | | F(4,31) = 6.177,  *p* < **.0001***** | 0.507 | | | 0.012 | | | -0.00 | | 0.011 | -0.029 | 0.016 | **0.028** | | | **0.01** | | F(3.32)=8.107  ***p* <.000***** |  |  |  |
|  | | **Intercept** | | **Age** | | **Gender** | | **Hearing** | | **STS** | | | | | | **Regression** | **Intercept** | | | | | | **Age** | | | **Hearing** | | **STS** | | | | | **Regression** | |  |  |
|  | **Estimation/**  **standard errors** | EST | SE | EST | SE | EST | SE | EST | SE | EST | | | | SE | | **Bonferroni:   *p* <** **0.002**** | EST | SE | | | | | EST | | SE | EST | SE | EST | | | | SE | **Bonferroni:**  ***p* <** **0.002**** |  |  |  |
| **WRITING PROCESS** | **Writing time in minutes** | *1968.7* | 362.4 | -103.3 | 308.9 | 227.0 | 247.7 | 73.1 | 367.0 | -115.3 | | | | 223.2 | | F(4,31) = 0.240,  *p* = .9134 | 1892.77 | 278.5 | | | | | 222.7 | | 243.9 | 79.38 | 361.4 | -114.6 | | | | 220.1 | F(3.32)=0.291, *p = .8316* |  |  |  |
|  | **Number of characters (linear text)** | *1070.9* | 388.9 | 445.5 | 265.9 | **765.5** | **331.6** | 722.6 | 393.9 | 353.3 | | | | 239.6 | | F(4,31) = 3.593,  *p* = .016* | 1633.5 | 323.0 | | | | | 477.4 | | 282.9 | 676.1 | 419.2 | 348.4 | | | | 255.3 | F(3.32)=2.654  *p = .*06524 |  |  |  |
|  | **Offline Writing flow (characters/seconds** | *0.747* | 0.139 | **0.190** | **0.10** | 0.060 | 0.118 | **0.285** | **0.140** | 0.116 | | | | 0.085 | | F(4,31) = 2.571,  *p =* .05728 | 0.791 | 0.107 | | | | | **0.192** | | **0.094** | **0.281** | **0.139** | 0.116 | | | | 0.085 | F(3.32)=3.422 *p = .02878** |  |  |  |
|  | **Online Writing flow**  **(characters/seconds)** | *0.890* | 0.152 | **0.251** | **0.104** | 0.117 | 0.129 | 0.251 | 0.154 | 0.130 | | | | 0.094 | | F(4,31) = 3.51,  *p = .0178** | 0.976 | 0.117 | | | | | **0.256** | | **0.103** | 0.244 | 0.153 | 0.129 | | | | 0.093 | F(3.32)=4.435  *p = .*01024* |  |  |  |
|  | **Transition time**  **median** | *1.98* | 0.39 | **-0.13** | **0.04** | 0.00 | 0.05 | **-0.13** | **0.05** | -0.04 | | | | 0.03 | | F(4,31) =5.144, *p* = .0026** | 1.977 | | | | 0.382 | | **-0.13** | | **0.035** | **-0.13** | **0.052** | -0.036 | | | | 0.032 | F(3.32)=7.075  ***p = .000****** |  |  |  |
|  | | **Intercept** | | **Age** | | **Gender** | | **Hearing** | | **STS** | | | | | | **Regression** | **Intercept** | | | | | | **Age** | | | **Hearing** | | **STS** | | | | | **Regression** | |  | 6.075 |
|  | **Estimation/**  **standard errors** | EST | SE | EST | SE | EST | SE | EST | SE | EST | | SE | | | | **Bonferroni:   *p* <** **0.002**** | EST | | | | | SE | EST | | SE | EST | SE | EST | | SE | | | **Bonferroni:   *p* <** **0.002**** |  |  |  |
| **PAUSES (1 sec)** | **Pause**  **percentage** | *68.52* | 32.05 | -0.86 | 2.95 | 2.76 | 3.69 | -0.23 | 4.38 | 0.26 | | 2.66 | | | | F(4,31) = 0.1561, *p* = .9588 | 69.31 | | | | | 31.8 | -0.75 | | 2.93 | -0.405 | 4.344 | 0.247 | | 2.645 | | | F(3.32)= 0.022  *p = .996* |  |  |  |
|  | **P-burst in number**  **of characters** | *4.88* | 1.38 | **2.78** | **0.94** | 1.63 | 1.17 | 2.26 | 1.40 | 0.24 | | 0.85 | | | | F(4,31) = 3.486, *p =* .01835*** | 6.08 | | | | | 1.09 | **2.85** | | **0.95** | 2.162 | 1.412 | 0.232 | | 0.86 | | | F(3.32)= 3.889 *p = .01775** |  |  |  |
|  | **P-burst**  **in seconds** | *2.02* | 0.38 | **0.69** | **0.26** | 0.43 | 0.33 | 0.39 | 0.38 | 0.07 | | 0.24 | | | | F(4,31) = 2.931,  *p* = .0364* | 2.34 | | | | | 0.30 | **0.703** | | **0.265** | 0.3626 | 0.392 | 0.071 | | 0.238 | | | F(3.32)=3.252  *p =* .0344* |  |  |  |
|  | **Number of pauses within words** | *414.18* | 201.0 | -22.7 | 18.53 | -42.92 | 23.11 | -37.3 | 27.5 | -6.70 | | 16.70 | | | | F(4,31) = 1.63, *p* = .1917 | 401.97 | | | | | 208.45 | -24.5 | | 19.20 | -34.64 | 28.46 | -6.42 | | 17.33 | | | F(3.32)=0.951 *p* = .4277 |  |  |  |
|  | **Pauses within**  **words in seconds** | *3.80* | 2.14 | -0.15 | 0.20 | 0.32 | 0.24 | -0.37 | 0.29 | 0.11 | | 0.18 | | | | F(4,31) = 1.293,  *p =* .2941 | 3.90 | | | | | 2.17 | -0.14 | | 0.200 | -0.391 | 0.295 | 0.108 | | 0.180 | | | F(3.32)=1.099 *p = .*3638 |  |  |  |
|  | **Number of pauses between words** | *238.23* | 254.43 | -4.09 | 23.46 | -12.67 | 29.26 | 10.37 | 34.77 | -3.52 | | | | | 21.15 | F(4,31) = 0.17, *p* = .9521 | 234.62 | | | | | 251.0 | -4.62 | | 23.13 | 11.14 | 34.27 | -3.44 | | 20.9 | | | F(3.32)=0.1685 *p =* .9169 |  |  |  |
|  | **Pauses**  **between words** | *3.68* | 2.74 | 0.07 | 0.25 | -0.03 | 0.31 | 0.36 | 0.37 | -0.31 | | | | | 0.23 | F(4,31) = 1.515, *p* = .222 | 3.665 | | | | | 2.695 | 0.070 | | 0.248 | 0.365 | 0.368 | -0.314 | | 0.224 | | | F(3.32)=2.08  *p = .*1224 |  |  |  |
|  | **Number of pauses between sentences** | *25.63* | 7.73 | **15.1** | **5.29** | -1.55 | 6.59 | 2.96 | 7.83 | -7.07 | | | | | 4.77 | F(4,31) = 1.515, *p =* .108 | 24.493 | | | | | 5.938 | **15.05** | | **5.201** | 3.052 | 7.707 | -7.063 | | 4.694 | | | F(3.32) =2.832  *p = .*05383 |  |  |  |
|  | **Pauses between sentences in seconds** | *56.17* | 32.12 | -4.08 | 2.96 | 3.32 | 3.70 | -2.75 | 4.39 | 0.24 | | | | | 2.67 | F(4,31) = 0.715, *p* = .5877 | 57.11 | | | | | 32.00 | -3.94 | | 2.95 | -2.949 | 4.37 | 0.22 | | 2.66 | | | F(3.32)=0.6066 *p = .*5656 |  |  |  |
|  | | **Intercept** | | **Age** | | **Gender** | | **Hearing** | | **STS** | | | | | | **Regression** | **Intercept** | | | | | | **Age** | | | **Hearing** | | **STS** | | | | | **Regression** | |  |  |
|  | **Estimation/**  **standard errors** | EST | SE | EST | SE | EST | SE | EST | SE | EST | | | SE | | | **Bonferroni:**  ***p* <** **0.002**** | EST | | SE | | | | EST | SE | | EST | SE | EST | SE | | | | **Bonferroni:   *p* <** **0.002**** |  |  |  |
| **REVISION** | **Removed**  **words in %** | *0.14* | 0.02 | 0.03 | 0.02 | 0.03 | 0.02 | **-0.05** | **0.02** | -0.00 | | | 0.01 | | | F(4,31) = 4.429, *p* = .006** | *0.164* | | 0.019 | | | | 0.027 | 0.016 | | **-0.052** | **0.024** | -0.002 | 0.014 | | | | F(3.32)=4.845 *p = .006849*** |  |  |  |
|  | **Removed**  **characters in %** | *0.15* | 0.02 | 0.02 | 0.02 | 0.03 | 0.02 | **-0.06** | **0.02** | -0.00 | | | 0.02 | | | F(4,31) = 4.164, *p* = .008** | *0.179* | | 0.020 | | | | 0.022 | 0.017 | | **-0.061** | **0.025** | -0.005 | 0.016 | | | | F(3.32)=4.422 *p = .*01038* |  |  |  |
|  | **Inserted**  **words in %** | *0.03* | 0.02 | **0.04** | **0.01** | 0.04 | 0.02 | -0.01 | 0.02 | -0.01 | | | 0.01 | | | F(4,31) = 3.368, *p* = .02119* | *0.055* | | 0.017 | | | | **0.037** | **0.015** | | -0.012 | 0.023 | -0.008 | 0.014 | | | | F(3.32)=2.941,  *p = .*04791* |  |  |  |
|  | **Inserted**  **Characters in %** | *0.02* | 0.01 | **0.02** | **0.01** | 0.02 | 0.01 | -0.01 | 0.01 | -0.01 | | | 0.01 | | | F(4,31) = 3.337, *p* = .02201* | *0.040* | | 0.012 | | | | **0.025** | **0.010** | | -0.008 | 0.015 | -0.005 | 0.009 | | | | F(3.32)=2.982 *p = .04857** |  |  |  |
|  | **R-bursts**  **in seconds** | *106.07* | 21.50 | **-7.68** | **1.98** | -2.47 | 2.47 | -4.18 | 2.93 | 0.58 | | | 1.79 | | | F(4,31) = 4.639, *p* = .00473** | *105.365* | | 21.48 | | | | **-7.78** | **1.98** | | -4.03 | 2.93 | 0.596 | 1.787 | | | | F(3.32)=5.853 *p = .*0026** |  |  |  |
|  | **R-bursts**  **in characters** | *44.86* | 16.65 | -2.88 | 1.54 | -0.20 | 1.92 | 0.53 | 2.28 | 1.71 | | | 1.38 | | | F(4,31) = 1.009, *p =* .4181 | *44.80* | | 16.39 | | | | -2.88 | 1.51 | | 0.542 | 2.237 | 1.708 | 1.362 | | | | F(3.32)=1.384 *p =* .2654 |  |  |  |
|  | **Major**  **revisions** | *0.17* | 0.04 | 0.02 | 0.03 | 0.01 | 0.03 | -0.06 | 0.04 | 0.01 | | | 0.02 | | | F(4,31) = 1.953, *p* = .1265 | *0.176* | | 0.029 | | | | 0.019 | 0.025 | | -0.058 | 0.038 | 0.010 | 0.023 | | | | F(3.32)=2.67 *p =* .06417 |  |  |  |
|  | **Global**  **revisions** | *0.01* | 0.27 | 0.01 | 0.02 | 0.01 | 0.03 | 0.01 | 0.04 | -0.02 | | | 0.02 | | | F(4,31) = 0.264, *p* = .8988 | *0.016* | | 0.262 | | | | 0.012 | 0.02 | | 0.009 | 0.036 | -0.017 | 0.022 | | | | F(3.32)=0.334 *p = .*8008 |  |  |  |
